# Adding an Online Community to an Internet-Mediated Walking Program. Part 2: Strategies for Encouraging Community Participation

**DOI:** 10.2196/jmir.1339

**Published:** 2010-12-17

**Authors:** Paul J Resnick, Adrienne W Janney, Lorraine R Buis, Caroline R Richardson

**Affiliations:** ^4^Health Services Research & Development Center for Clinical Management ResearchVeterans Affairs Ann Arbor Healthcare SystemAnn Arbor, MIUnited States; ^3^College of Nursing – Adult HealthWayne State UniversityDetroit, MIUnited States; ^2^Department of Family MedicineUniversity of MichiganAnn Arbor, MIUnited States; ^1^School of InformationUniversity of MichiganAnn Arbor, MIUnited States

**Keywords:** Social support, online community, Internet, adherence, retention, exercise, health, forums, on demand, support

## Abstract

Starting a new online community with a limited number of members who have not self-selected for participation in the community is challenging. The space must appear active to lure visitors to return; when the pool of participants is small, a large fraction must be converted from lurkers to contributors, and contributors must receive responses quickly to encourage continued participation. We report on strategies for overcoming these challenges and our experience implementing them within an online community add-on to an existing Internet-mediated walking program.

Concentrated study recruitment increased the effective membership size. Having few conversation spaces rather than many specialized ones, staff seeding of the forums before members were invited to visit, and staff posting of new topics when there were conversation lulls, all helped to make the forums appear active. In retrospect, using even fewer separate spaces and displaying a flat rather than nested reply structure would have made the forums appear even more active. Contests with small prizes around participation in the forums and around meeting walking goals generated a lot of discussion; a contest for first-time posters was especially effective at moving lurkers to post. Staff efforts to elicit participation by asking questions had mixed success. Staff replies to posts that had not received member replies created a feeling of responsiveness despite limited membership.

## Introduction

Starting a new online community with a limited number of members who have not self-selected for participation in the community is challenging. This paper reports on design, management, and moderation strategies for overcoming these challenges and our experience implementing them within an online community add-on to an existing Internet-mediated walking program.

Online communities are everywhere on the Internet. People who share an interest in a hobby, a product, a political cause, or a celebrity join in conversation [1]. Those who share a problem or a solution to a problem find each other on the Internet as well. In health-support communities, people share disease-specific information as well as provide support and encouragement [2-7]. Yahoo! Groups, just one of many available platforms, claims to host millions of groups.

While there are many success stories, there are even more that have failed to take off. For example, Butler found that a third of public hobby and work mailing lists had no traffic over a 4-month period even after significant screening to eliminate nonfunctioning lists [8]. Efforts to test the impact of online communities on participants are a particularly risky research enterprise if they require generating a new community. For example, smokefree.gov, an online tobacco cessation program, attempted to add an online community for some of its users but was unable to garner enough activity in the community during the trial period to determine whether such a community, if it were active, would help users quit smoking [9]. Presumably, many more failed attempts to create online communities in research settings go unreported.

It is especially difficult to create a new online community as a support to some other program or activity that has a limited pool of potential members who have not self-selected for online community participation. Such settings include communities of practice within small organizations, discussion forums associated with courses, and medical interventions where only participants in the research study are eligible to participate in the online community. In some settings, such as courses and medical interventions, a limited duration for the community or for individual participation in it (16 weeks from joining in our case) may pose an additional challenge by reducing the opportunity for interpersonal bonds to form, requiring a greater dependence on commitment to the group or the activity as a whole to motivate participation [10].

Not everyone who visits a Web-based community or becomes aware of an email list will participate at all, even as a lurker. In arenas such as consumer product support, where every customer is a potential member, simply getting enough visitors can be enough to kick-start active discussion. One provider of product-support communities estimates that in any given month, 10% of visitors to a product website will follow a prominent link to discussion forums, and 10% of these will post [11]. Thus, 5000 monthly visitors to a product website could be expected to yield 50 posters, which would be sufficient to generate active forums if a few of the posters were to become regular contributors. Nonnecke and Preece found that just less than half of subscribers to health-related email lists lurked without posting, and more than 80% of subscribers to software support email lists did so [12]. As they point out, this is not necessarily a problem, since lurkers gain value from reading, and posters may gain value from having an audience. When the pool of eligible participants is much smaller, however, it is necessary to attract a larger percentage to post to create enough content to keep people coming back.

Once people post, the reaction they get can help decide their continued participation. Previous studies have shown that first-time posters who receive a response are more likely to post again [13] or to post sooner [14]. In the largely technical community Slashdot.org, the valence of the reaction did not seem to have an effect; continued participation depended merely on whether the poster received a response at all. In a health-support community, however, it seems likely that responses that provide requested information and are emotionally supportive will be more effective at encouraging additional contributions.

In all, 3 major challenges arise, then, in building a new online community, especially with a limited pool of potential members and a limited time horizon. The first is to present the appearance of an active space that has interesting content and people with whom to interact so that visiting members will want to keep coming back. The second is to convert members from lurkers to posters. The third is to ensure that posters receive appropriate responses.

Implementing an online community involves a variety of strategic design choices about software configuration, about activities and conversation topics to introduce, and about types and quantity of staff participation. These strategic choices can have a big impact on the success or failure of an online community. Prior research has investigated design choices and behavior in mature communities [5,15-25]. Researchers developing new ways for people to interact have conducted empirical assessments by forming new user communities, but their reporting has not focused on the process of starting the new communities [26-28].

Stepping Up to Health (SUH) is an Internet-mediated walking program designed to collect walking data and return feedback to the user to produce a gradual increase in walking. Participants receive a pedometer to record step counts, which they upload periodically over the Internet. The main page of the website features a graph displaying step counts against goals as well as some textual feedback about walking progress, tailored motivational messages, and tips about walking. In this iteration of SUH interventions, some participants also received access to an online community through the SUH website.

The online community was successful at encouraging retention in the program (21% vs 34% dropout rate). Participants in both arms increased their walking significantly, with no difference between the arms. The companion paper, Part 1, gives more details on the aforementioned results [29]. While in Part 1 we examine differences in outcome between community members and nonmembers, as well as explore potential mechanisms for differences, here in Part 2 we report on the choices made when adding discussion forums to an SUH intervention and reflect on their impacts on member participation in the forums.

### The Community

**Figure 1:**
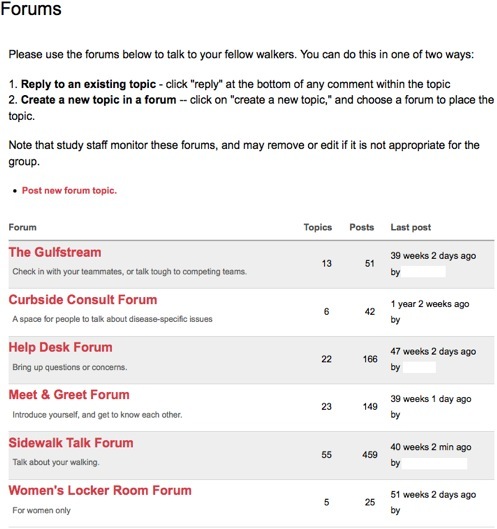
Available forums in the online community

The online community component added to Stepping Up to Health was implemented using the forums module of Drupal, an open-source content management system. Only the 254 intervention participants randomized to the online community arm were able to access the forums. We will refer to these participants as the *members* of the community even though not all of them chose to participate in the community itself. Members could see a link labeled “Talk to other participants” in the left sidebar menu, which took them to a page showing the available topical forums (see Figure 1). In addition, members could scroll down the initial log-in page to see teasers for the 5 most recently active post titles in the online forums. Members could also fill out profiles and read each other’s profiles.

### Posting

**Figure 2:**
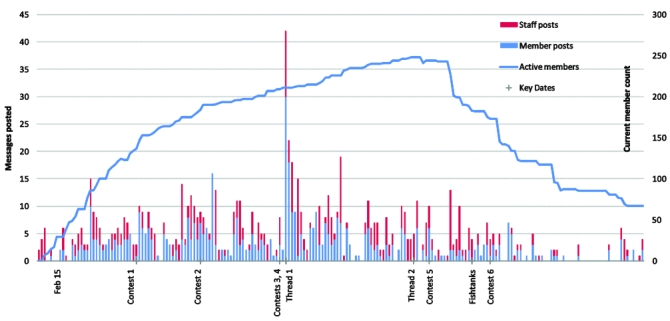
Timeline of interventions and participation

As shown in Figure 2, after an initial start-up period and until members who had completed the program started losing access to the forums, on most days there were 3 to 10 posts. Participants posted 56% (524) of the 929 total forum messages, with staff posting the rest. Of the 254 people assigned to the online community arm, 114 (45%) posted at least once, 22 (9%) posted more than 5 times, 12 (5%) posted more than 10 times, and 1 member posted more than 50 times. Those who posted averaged 5 posts per person (median 2).

### Viewing

Participation in an online community can be passive as well. Of all 254 members, 52 (20%) were lurkers, defined as never posting but viewing an average of at least 1 forum page per week (16 pages over the course of the study). Members viewed pages in the online community at varying frequencies, with a median of 24 views; 4 members viewed more than 1000 pages. Those who viewed more pages posted more messages (Poisson regression, *r* = 0.65, *P* < .001). Of all members, 5% (12/254) never viewed a forum page.

### Content

No malicious or inappropriate posts appeared on the site. The most popular topics were discussions on walking motivation and strategies, physical health, and study procedures. Table 1 contains a detailed breakdown of post content.

**Table 1:** Post content, all posts

Category	Percent of All Posts (929)
Strategies/motivation	58.1
Physical health	15.9
Study procedures	11.8
Diet/nutrition	11.1
Other	10.4
Pedometer	10.0
Website	6.5
Mental health	5.7
Teams	5.7
Introductions	4.3

As with other online health communities, posts offered a mix of information and emotional support [5], and some members took inspiration from others’ successes.

Examples of member posts on motivation and strategies include:

In the past, I looked for the closest parking spot; now I find the farthest and it helps to add steps for the winter walks.

…Because of the weather for now my walking is daily steps plus getting on the treadmill in the evening. I’m hoping that once the weather gets a little less muddy I am going to go out and explore my woods. I agree it helps to have a partner to motivate you. I used to have a friend who lived close to me and we would walk a few times a week. Now we are too far apart for that to be convenient.

The following is an example of a post that broaches physical health issues:

My feet and back had been “uncomfortable” after lots of walking. I hesitate to say hurting, but more like tired and sore. Then I realized how old and probably broken-down my tennis shoes were and after buying new tennis shoes specifically for walking and new inserts that provide arch support, I’ve found it a pleasure to walk again.

Participants congratulated and encouraged each other in some posts:

Congrats on your loss of 6 lbs in 5 weeks! Do you have any tips? I seem to want to eat more, not less. I’ve only been maintaining at best.

Congrats on completing a full week and keep up the great work.

I know I have only one full week of completion on my record as well. I allow myself one day a week to not worry about the daily goal. I do, like you, make sure to keep my daily average above the goal, ie, I make up the steps during the week. As a matter of fact I just had my largest one-day total tonight…

Some posts showed explicit evidence that members took inspiration from each other:

There I was on Saturday night sitting around and came across your messages on 100K. I realized I was in reach, so I grabbed my walking poles and left the house at 9pm Saturday just to say I could do it. Thanks again.

Some interpersonal bonds were formed, as evidenced by posts that announced and acknowledged the impending departures of certain members. For example, a new topic and the first reply:

I've completed my 16-week participation in the study, and I've been informed by the staff my account will be deleted this weekend. Therefore this will be my last opportunity to log in to the site and post a message. I'm going to use this opportunity to say farewell to all my friends here. To those of you who have walked along with me for weeks and will be completing participation yourselves soon, my best wishes to you for continuing success walking…

Good luck with keeping up with your walking with your new lifestyle! You are a great motivator and will be missed. Just think for a minute that walkers are reading your posts, getting motivated, and just taking it all in. You have accomplished a lot and should be very proud of yourself!

### Design Choices and Their Impacts

While the online community effectively provided information and support, its success was not entirely organic. Staff authored 44% (405/929) of all posts and made numerous strategic choices regarding the design and management of the community. We present those choices, describe their effects, and make suggestions for future community designers. The narrative is organized around the 3 challenges that the design and management strategies were intended to address: presenting the appearance of activity, motivating lurkers to post, and assuring responsiveness.

### Presenting the Appearance of Activity

The appearance of inactivity can create a self-fulfilling prophecy. If a member checks the forums and finds nothing new and interesting, he or she might form an expectation that nothing much happens in the forum and not bother to check it again. One vendor offered a rule of thumb: a forum needs 5 to 10 messages per day to feel active enough to spur ongoing use [11].

#### Concentrated Recruitment

Our first strategy for presenting the appearance of recent activity was simply to maximize the number of members who could potentially be active. As described in the companion paper, to create a more active community, the randomized trial employed an imbalanced design with more people in the online community arm. In addition, we tried to concentrate recruitment into the study to create as much overlap as possible in participants’ 16-week participation windows. This required a novel recruitment strategy for the intervention. We abandoned traditional clinical trial recruitment practices, such as fliers in hospitals, clinics, and public places, which have low yield. Instead, we pulled a list of potentially eligible patients from the clinical data warehouse of a large medical system and mailed targeted recruitment letters to these individuals. For a detailed report of recruitment results, see Part 1. We also moved screening and enrollment to the Internet; without the need for face-to-face encounters with staff (though often with significant phone and email support), we were able to process participants in larger waves.

Even with these efforts, it was not possible to synchronize the start—and thus the end—dates of members fully. Participants took varying amounts of time to complete the prerandomization requirements: medical clearance, online survey, and uploading baseline step-count data. Once participants had completed all the preliminaries, we were worried that making them wait before starting the walking intervention and the online community participation would demotivate them.

Finally, we did not have sufficient staff to handle intake of all participants simultaneously, even with a largely automated process, so we sent out invitation letters in waves. Thus, we still had staggered start dates. The line graph in Figure 2 shows the number of members who had access to the forums during the period of the study. Not surprisingly, as shown in the blue bar graphs in Figure 2 (member posts), the greatest participation in the forums also coincided with the period in which the greatest number of members had access to them.

#### Few Separate Forums

Before the experiment, it was easy to imagine many different things that members might want to discuss. It was tempting to create a separate forum for each category of topic, both to suggest the different kinds of topics to members and to allow them to navigate to just the ones they found interesting. For example, we wanted to provide a place where people could discuss technical support questions with respect to the pedometer or uploading functions without intruding on discussions about motivations for walking. Because there are gender-specific barriers to exercise, we also wanted to provide separate single-sex forums where men could interact with men and women with women.

We anticipated, however, that we would not have enough conversation to keep lots of different forums populated with new content. Thus, we limited the initial set of forums that anyone would see to 5. Figure 1 shows the initial 5 forums that were visible to women plus “The Gulfstream,” which we added later for team competitions, described below. Men saw “Men’s Locker Room” instead of “Women’s Locker Room.” In retrospect, we probably should have been ruthless in limiting the number of forums. The Men’s Locker Room had only 7 threads, the Women’s Locker Room, 5, and Curbside Consult had only 6. Visitors to any of these specific forums would have found no recent conversation in them. Having just a single forum might have been the most effective way to avoid the possibility of members encountering dead zones with no recent activity.

#### Flat Versus Nested Display of Conversations

There are two common display formats for online discussions. The first, known as a “flat structure,” displays comments in chronological order, with either newest or oldest first. Flat comments are common in blogging packages and online newspapers and magazines. While comments follow a particular story or post, in a flat structure the display does not indicate which comments are replies to others, so writers sometimes name the author or otherwise describe the comment to which they replied. The second format, known as “threaded” or “nested,” is more often used in discussion forums. Replies usually have an indent or other visual marker to set them apart from new comments. Each comment has a reply option, and writers choose the appropriate place to insert their messages, possibly in the middle of the displayed page.

Since we envisioned our online community features as discussion forums, we used the threaded display. One drawback of this structure, however, is that since the newest messages may be in the middle of a conversation thread, it is possible for a discussion to look stale to a first-time visitor even if it is not. Moreover, some of our users were not very familiar with discussion forums and did not realize that it mattered which button labeled “reply” they clicked on, and so some messages appeared indented under other posts they were not in fact replying to, which made it confusing for readers. Finally, unlike some discussion boards, ours contained no demarcation of posts unread by a specific user, so members could not hunt for replies to their posts without remembering where they had posted and then navigating back to them. We suggest that other designers of online communities for people who are not already experienced forum users would do better to select a flat display rather than threaded and possibly use software that allows for an individualized notification scheme.

#### Initial Forum Seeding and Restarting Conversations

Staff seeded the forums with initial content so that the first members to visit would encounter a nonempty space. As shown in Figure 1, members could see how recently content had been posted to each forum. To convey the sense of a lively space, we delayed adding the staff-seeded content until the week when the first members received access to the online community.

Overall, we seeded 12 posts into the forums before members arrived. Of the seeded posts, 8 contained staff introductions, and 1 post introduced each of the other initial forums. Of the initial posts, 7—4 of the personal introductions and 3 of the forum introductions—explicitly asked questions or invited members to post information.

To convey on any member visit the impression of recent conversation, staff monitored the forums and started new discussion topics whenever there was a lull. Staff started 75 of the 133 total topics in the forums.

### Encouraging Posting

A second challenge in a forum that has only a few members is to coax as many as possible to post rather than just reading. We made 3 design choices aimed to increase member posting: questions, posting contests, and walking contests.

#### Questions

First, many of the staff-initiated threads and staff responses to member posts employed the rhetorical ploy of asking questions. When answering a member’s question, the staff member would also ask the member a follow-on question or encourage additional responses from other participants. For example (emphasis added):

I know when I get home from work, my first instinct is to veg out or do things around the house. It helps me if I make plans with a friend to go exercise. **Do you have anyone, in your household or outside it, who might want to make a walking date with you?**

…[Information about preventing blisters and shopping for shoes, responding to a member concern with blisters]… I hope some of this information helps. Let us know what works and what does not work for you. **I’m guessing that there are others who are in the Stepping Up to Health program who have experienced blisters also. We can learn [from] each other in the forums.**

Staff reported that they sometimes felt they had overused this rhetorical ploy. Results were somewhat mixed. Of 39 staff responses that posed a question back to the original poster, only 12 elicited a response from the original poster, and 6 elicited a response from someone else. Staff responses that explicitly solicited replies from the whole community were somewhat more effective: Of 19 such messages, 5 elicited a response from the original poster, and 10 elicited a response from someone else.

Initial posts that asked questions as a way to generate conversation were more effective. Some introduced topics that many people could relate to and contribute to, such as vacation plans or the following post on coping with mosquitoes, which generated 14 responses.

I'm very happy that it's summer, but I've heard a lot of complaints from coworkers about the ravenous mosquitoes…that appear around dusk as well as the clouds of gnats that seem to appear late afternoon everyday. These little friends can really take the enjoyment out of an evening bike ride or walk. Does anyone have some good suggestions on how [to] overcome this natural obstacle to a relaxing evening walk?

Bugged out…

Another successful conversation starter was a personally revealing anecdote accompanied by a request for suggestions. Personal revelations are known to increase interpersonal attraction in laboratory settings [30]. The role reversal of having someone who usually provides support instead asking for it can also serve as an icebreaker.

My baby sister graduates this weekend! Because I love my little sister, and I'm very proud of her, I'm going to her graduation. But in making the plans I realized something—it's really going to mess with my exercise schedule. Anyone else having this problem? Time sitting in the car, time sitting at the graduation, time sitting in restaurants…Plus switching my gym time around so I could add a whole bunch of graduation stuff to my weekend will leave me at least one planned workout short this week. Anyone have ideas for how to get some walking in at times like these?

Members responded by completing the role reversal, not only providing tips, but also suggesting that she rethink whether she was getting too obsessive about her exercise. The thread generated 16 comments in all. Moreover, members asked questions of their own in response, as well as giving advice to the staff moderator. A sampling of 2 of the member responses follows:

I guess when I read that I could totally relate and that is why I am hoping you are not offended when I say it sounds a bit obsessive. I just said to my friend today that...“My husband wants to meet me for lunch today, but if I do that I won’t get my walk in.” I guess I am answering your post with another question…[Do you think activity] begins to feel not so much like something just to do, but something you have to do? I have been struggling with that as I would like to lose some weight but I am feeling a bit deprived of the “carefree-ness” of not paying attention to everything I eat and how much I walk.

So I wonder, [name redacted], if the question you are really asking is not “is it OK to skip this walk so I can see my baby sister graduate [?]” but “have I reached that state of confidence and balance that tells me I'm in control, so I won't worry about swapping my sister's graduation for a walk?” We all have to get from counting steps to counting on ourselves somehow. How do we get there from here?

#### Posting Contests

A second strategy for increasing member posting was contests. The contests were time-limited, and all but 1 of the 6 centered on posting.

The first contest came about a month after the forum opening with more than 100 members able to access the forums and promised members who posted that day or the next that their post would be entered into a “staff favorite” judging. The winner would receive an unspecified prize in the mail. The contest announcement produced 42 responses.

The prize for the first contest was a water bottle. Small monetary rewards can have a demotivating effect [31], but the low-cost prizes were a hit. The staff picked 2 winners, and both posted about their prizes without revealing what they were.

I wanted to let you know the award package arrived in the mail on Friday without having been broken, flattened, eaten, stained, spindled, or creased by the postal service…Everyone will just have to trust me that they will want to win. Anyway, my thanks to the staff for selecting my posting as one of the winners. That won't stop me from trying to do better in the next contest (if there is one) either.

The second contest, specifically intended to get lurkers to unveil themselves, took place about 3 weeks after the first. Anyone posting for the first time within this 5-day window was eligible for a prize drawing. The thread generated 26 responses.

The next contest, about a month after the second, invited members to post a favorite healthy snack idea. In all, 45 members posted snack ideas on the thread and again were eligible for a single-winner drawing. Staff compiled and grouped the snacks in a new thread that received only 1 reply.

The final posting competitions took place 6 and 9 weeks later, respectively. Both occurred as participants were exiting the forums, and both were repeats of previous contests: the “staff favorite” and the “first-time poster.” Table 2 shows each forum event, including contests, and the number of replies generated.

**Table 2:** Index of staff interventions

Date	Event Type	Event	Number of Replies Generated
Mar 12	Posting contest	Contest 1: Staff favorite	42
Apr 2	Posting contest	Contest 2: Virgin poster	26
Apr 29	Posting contest	Contest 3: Healthy snacks	46
Apr 29	Walking contest (individual goals)	Contest 4: Meet own walking goal 5 of 7 days	37
May 1	Seeding thread	Thread 1: Role reversal, with advice and support to staff	16
Jun 12	Seeding thread	Thread 2: Mosquitoes	14
Jun 17	Posting contest	Contest 5: Virgin poster	9
Jul 1	New feature announced	Fish tanks introduced	9
Jul 7	Walking contest	Team competition announced	2
Jul 7	Posting contest	Contest 6: Staff favorite	4

#### Walking Contests

A third strategy was to create common experiences in the walking program that became foci for conversation in the online community. Staff announced a contest to meet one’s personal walking goals 5 out of 7 days in a particular week. Rather than draw from a hat, the staff sent an “I (heart) walking” bumper sticker to each person who met the criteria. The contest thread generated 37 replies.

In a similar vein, toward the end of the intervention on July 1, 2008, we introduced an element of team camaraderie and interteam competition. The earliest participants had already completed their 16 weeks in the program and no longer had access to the forums. We assigned remaining members to 10-person teams and added a new forum just for discussion of team competitions. Drawing on the Tamagotchi-like idea of feeding a pet fish through one’s exercise that had proved effective in a different walking intervention [32], we showed a graphic of a fish tank. Each fish represented a particular member, and a fish’s visible health (color, movement) represented the member’s walking progress. We announced the competition a week later, with T-shirt prizes to members of the team that collectively met the highest percentage of their members’ goals.

While some of the features of the team assignment and fish tank display received mixed reviews from participants, they did generate a flurry of messages trying to generate team spirit. The following exchange was typical:

I am alive and well!! Lost my pedometer but I am back now! Getting some color. Let’s go for a swim!

Hey [name redacted]. Glad to have you back and in color! Wish we could help a few of those grey fish in our tank! But I’m happy for you! Swim on!

### Responsiveness

Newcomers to online communities who receive a reply to their first post are more likely to post again [13,14] or to post sooner [33]. More generally, we thought that the forums would feel more responsive and thus invite more participation if all posts received responses. Our strategy to achieve responsiveness was to have staff reply whenever members did not.

Staff logged into the forums most days, looked for posts that had not received responses, and responded to them. Overall, all but 3 of the 58 member-initiated threads received replies, either from other members or from staff. The 2 threads that did not receive replies were a post addressed solely to team members in the team walking contest and a staff oversight. The median time to first reply was 11.2 hours, and 46 out of the 58 threads received a reply within 24 hours.

Staff also made an effort to respond to member posts that did not start threads except for those that were simple offers of support or encouragement. Of the 466 member comments that did not start new threads, 12% received replies from other members, and 36% received replies from staff, with a median time to first reply of 19.3 hours for those that did receive a reply.

Staff responses, like member posts in health forums more generally, included 1 or more of 3 different kinds of content. Of these, 1 type provided information and advice, such as:

To avoid unhealthy heat and UV ray exposure, I would encourage outdoor walkers to get their outdoor activity in before 10am and avoid strenuous activity until after 4pm. If the temperature is hot and the humidity is high, be sure to bring along some ice-cold water in a water bottle and wear lightweight clothes and appropriate sunscreen. If you can choose your outdoor walking routes, why not select routes that are shady and take you by pleasant gardens, wild flowers, and other scenic summer foliage?

Another category of content was emotional support, including encouragement, reassurance, or thanks to the poster. The supportive response could be either related to the physical activity intervention or to participation in the forums. For example:

Wow! I love that idea. That is an excellent idea as a reward for finding a way to fit walking into your day.

Congratulations on doing so well with your walking goals, and thank you for sharing your progress with everyone within the forums…

A third category of content was reflections and anecdotes about the staff member’s own barriers or approaches to physical activity. Such posts validated members’ struggles, and we expect that they were perceived as emotionally supportive even when they did not directly provide any suggestions or encouragement. Following are 2 examples of such posts:

My family just got a new puppy…He is a handful and chews up everything in the house but he definitely gets us out of the house more often and he definitely helps me keep my step counts up. And he is pretty much always up for another walk if you need a walking buddy...

Not being very active when working is a problem I face a lot. Here are some of the strategies I use to get a little more active…

## Discussion

We made two design decisions that helped to concentrate activity so that visiting members did not find an empty space. First, we altered our recruitment methods to ensure that as many people as possible would have simultaneous access to the online community. The first burst of posting (10 messages in 1 day) came after 2 weeks following a burst of 23 new members in 2 days, which brought the total membership to 86. This suggests that even more concentration of entry into the community probably would have helped it to take off faster. Second, we limited the number of initial forums to 5 to make it less likely that members would encounter forums without recent activity. Deciding which 5 to include was a difficult process that required jettisoning personal favorites of some staff. In retrospect, we probably would have done better simply to group all of the conversation into a single forum so that members would not need to navigate to multiple pages to find all the new posts.

We employed several seed-and-feed tactics to elicit more participation from members than they might have contributed organically. We seeded the forums with initial content to lower the burden of coming up with a topic for first-time visitors. This seemed to be less successful than we had hoped, however, as the members made only 16 posts in the first 2 weeks in response to those seed messages. We posted new threads whenever we sensed a lull in the conversation. We went out of our way to make sure that any member posts where a reply was appropriate received one. We employed the rhetorical tactic of asking questions in our responses, though that met with mixed success. We also employed a rhetorical tactic of having the staff relate personal anecdotes, which often elicited replies from members and, on rereading the forums after the study, seems to have created a warm, personal feeling that may have set a positive tone for member interactions.

Staff did not separately track their time devoted to 405 posts worth of seeding and feeding, but we offer a rough estimate. We estimate 1 hour of staff time to compose each of the 75 messages that started threads, including some that were carefully crafted in multi-person staff meetings. We estimate 15 minutes to compose each of 330 staff response messages, averaged over those that were short and those that were longer and required research. Finally, we estimate 10 minutes of staff reading time for each of the 524 member messages, since multiple staff followed the posts in the forums. The total is just under 245 staff hours, or the equivalent of about 6 weeks full-time for 1 staff member. While it was a significant effort, it was, for example, probably smaller than the amount of effort that went into designing, implementing, and testing the additional online community features that were added to the original SUH walking intervention. In many situations, this level of staff involvement would be reasonable.

One danger in providing staff contributions to make up for those that members might provide in larger communities is that staff content may drive out contribution of the members who are present. We do not have a way to estimate the extent to which such undesirable substitution occurred.

The occasional contests, with unspecified token prizes, were the most effective single intervention at producing participation. The 4 most popular threads were all prize threads. The most popular of these started a new topic that was of great interest to the participants (walking and snacking), but even the contests for first-time posts and for unspecified good posts on any topic were effective at eliciting participation. The contests were largely noncompetitive in nature, since there was no visible means of comparing anyone’s performance with others’ with the exception of the “meet your goal 5/7 days” contest, where some members offered “I made it” posts, and others seemed to be discouraged by not making it. We recommend that other online community managers consider the use of contests as a low-cost and effective way to generate participation, especially contests that reward participation over performance.

Finally, we found that team competition in the underlying activity (walking) tended to generate “go team” messages in the forums. Our team competition came late in the study, when many members had already completed the 16-week program and thus no longer had access to the online community. In addition, our implementation was imperfect. Even so, more than 5% of all the posts in the forums for the entire length of the study were about the team competition and the team fish tank visualization. Team competitions may not be available as a design option for all online community managers, since there may be no underlying activity on which teams can compete. Moreover, managers should employ them with caution, as some people may have a negative reaction to competition even though many others will not.

### Conclusion

Our major conclusion is that with enough careful design and staff effort, it is possible to create an online community on demand that is sufficiently active to retain participants, even with a small number of temporary members. A number of design choices are available that will increase the density and timeliness of participation. Seeding-and-feeding tactics can substitute staff participation for what a larger number of members might provide naturally and elicit more participation from the members who are there. The most effective single tactic we found was contests with small prizes for posting in the forums.

We have reported on a single case study. We have attempted to document and reflect on the design decisions we made. They are not, however, sufficiently transferable and actionable to guarantee that the results can be reproduced in other settings, especially given that the effects of individual members of online communities may have large effects on community outcomes.

While a more scientific test of the effectiveness of different community designs and management tactics would come from controlled experimentation, it would be prohibitively expensive to start a large number of new online communities. Moreover, in most settings, if there are enough people to form many online communities, the network effects would make it even more effective to form a single, larger community. Thus, case studies, with careful documentation of design choices and management tactics and their apparent impacts, are likely to be the best way to accumulate knowledge about how to start online communities. We hope to see many more such case studies of the formation of new communities.

## Abbreviations

SUH: Stepping Up to Health
